# Editorial

**Published:** 1860-08

**Authors:** 


### EDITORIAL.

*Diphtheria.*—Having received several letters from physicians in different sections of the country, stating that Diphtheria was more or less prevalent, and asking our opinions in relation to the treatment of that disease, we have thought it best to make some observations on that subject instead of the usual clinical reports. So far as experience has enabled us to form an opinion, we regard the Diphtheria as an affection intermediate between Scarlatina and Membranous Croup. Like the former, it usually commences with fever, but resembles the latter in the almost constant tendency to form false membrane, or at least curdy exudations upon the surfaces involved in the disease. Like the former, again, the fever seems constantly prone to assume an adynamic or typhoid grade of action, with a degeneration of the fluids, as indicated by the offensiveness of the secretions, especially from the mouth, fauces, and nostrils. In its sequala, also, it closely resembles scarlet fever, often leaving the patient anemic, with chronic suppurative inflammations of the mucous membranes, and lymphatic glands. On the other hand, it not only resembles membranous croup, in the tendency to plastic or fibrinous deposits on the inflamed surfaces, but also in its disposition to attack the larynx. From these facts, some writers have regarded the disease as only a modified form of inflamatory croup, while others have claimed its identity with scarlatina. The larger number, however, regard it as a distinct disease, but closely allied in its pathology to both the others named. It is not necessary to occupy either time or space with a detail of the symptoms of diphtheria, to accomplish our present purpose.

It is sufficient to state very briefly our views of its pathology, and see how far a rational treatment can be founded on them. The disease involves primarily, an alteration in the composition and properties of the blood, with a perverted state of that property which we designate vital affinity in the organized textures, and the development of a local inflammation in the glands of the neck and mucous lining of the fauces.

The special changes observable in the composition of the blood, consist in the diminution of red-corpuscles and sometimes of the albumen; fibrine is much increased. The diminution of the two first is accompanied by a corresponding depression of vital activity, while the increase of the latter coupled with a perverted affinity, causes the ready exudation of a fibrinous layer upon the inflamed mucous membrane of the fauces, and often also upon cut or abraded surfaces in any part of the body; and it is doubtless a copious infiltration of the same fibrinous material into the texture of the tonsils and lymphatic glands that causes them to become so swollen and hard early in the progress of the disease. At a former period, the appearance of an excess of fibrine in the blood was universally regarded as an evidence of a sthenic or *actively* inflammatory condition, that constituent being regarded as one of the nutritive elements of the blood. But more than ten years since we satisfied ourselves by observations and direct experiments that it was a product of disintegration and consequently excrementitious, being chiefly eliminated by the kidneys. Hence instead of regarding its presence in excess, as an evidence of a sthenic state of the system with exaltation of the vital forces, we look upon it as simply indicating, either a too rapid disintegration of nitrogenous structures or a failure of the kidneys and skin to eliminate it with the usual rapidity, or both these conditions co-existing at the same time. Conceding these views to be correct, there is no difficulty in explaining the co-existence of blood containing an excess of fibrine and a strong tendency to fibrinous deposits, with a perverted and depressed condition of the properties of the organized structures; thereby presenting all the phenomena of debility, depraved secretions, and a typhous tendency, together with excessive fibrinous deposits and exudations. And such really seems to be the exact pathological condition and tendencies in the majority of cases of well marked Diphtheria. It is true that we have seen some cases that have presented a full pulse, hot skin, and throughout a higher grade of action, resembling the ordinary inflammatory croup. But these are rather exceptions to the general rule.

*Treatment.*—The objects desirable to accomplish in the treatment of Diphtheria, are: 1st, to restore the normal condition of the blood by increasing the solubility and excretion of the fibrine, and thereby remove the tendency to further pseudomembranous deposits in the fauces or elsewhere. 2nd, to correct the perverted properties of the solids and thereby restore a more healthy secretory action generally and arrest the febrile movement. 3rd, to mitigate the local inflammations.

If it is true, that fibrine is a product of disintegration or metamorphosis of the tissues, its accumulation in the blood may result from two causes; first, a too rapid metamorphosis or waste of tissues, as we see in all the more active local inflammations; second, a failure of the kidneys and the secretive organs to eliminate it as fast as it is formed. Hence to fulfil the first object in the treatment, we use such agents as will hasten the oxydation and excretion of the fibrine, without reducing the strength of the patient. For this purpose we give pretty freely, internally, the chlorates of Potassa and Soda, and if the disease advances with fœtid secretions from the mouth and nostrils, we add the Muriated Tincture of Iron. To fulfil the second indication in the treatment, namely, to restore the vital properties to a normal condition, and thereby re-establish healthy secretion and remove the general febrile symptoms, we have been in the habit of giving, during the first day of treatment, between the doses of chlorate of Potassa, Calomel and Dover's Powder in alterative and anodyne doses, and follow them on the second day by a mild laxative, simply sufficient to move the bowels. After this we give, alternated with the chlorates or the Muriated Tincture, the following:

℞ Nitrous Ether, ℥ iss.

Tinct. Gelsemin, ℥ ss.

Tinct. Belladonna, ʒ i.

Mix, and give from 10 to 30 drops, according to the age of the patient, every three or four hours. The Gelsemin and Belladonna allay the general morbid excitability of the system, while the Nitrous Ether increases the eliminations from the skin and kidneys. To meet the third indication, *i. e.*, to counteract the local inflammation, we use a variety of local applications. In the first stage while the glands of the neck are swelling more or less rapidly, we keep the whole exterior of the neck thoroughly fomented with an infusion of Aconite leaves, holding in solution Muriate of Ammonia.

After the first day or two we change this for a liniment of Olive Oil ℥ ii., Oil Turpentine ℥ ss., and Chloroform ℥ ss., mixed and applied to the neck every two or three hours. In the first cases of the disease, that came under our care, we applied Nitrate of Silver and other active agents to the fauces and throat, but we did not derive that advantage we anticipated, and soon ceased using anything of the kind. We believe the solution of the chlorates and the diluted tincture of Iron, swallowed as internal remedies, come fully in contact with the fauces and constitute the most useful local applications—whenever the peculiarity of breathing and cough indicate the extension of the local inflammation to the Larynx, constituting the symtoms of *Croup*, we promptly give an emetic dose of Sub-Sulphate of Mercury (Turpeth Mineral)—and if necessary, repeat it every two or three hours until those symptoms are relieved. We had intended to add some further details in regard to the treatment of Diphteria, but as the Printer is after copy, we will simply append the following letter received a few days since from Dr. Slack, of Indiana:

" Diphtheria here for the last six months. I have found no difficulty in curing every case, when called in the start. I saw three cases that had been treated by other physicians, that proved fatal. I have only lost one case where I was the only one called, and that one only lived twelve hours after I first saw him. This disease is almost exclusively confined to children from two to ten years of age. I suppose I have treated over twenty cases, and when first called I generally find them making very little complaint. Examine their pulse, and I generally find very little excitement of the arterial system. Get them next to protrude their tongue, and it is almost invariably coated with a light yellow coat; next press the tongue down with a spoon handle, and you will see one or both tonsils enlarged, and in the centre of one or both, as the case may be, an ash colored ulcer or false membrane. The bowels are generally costive. I generally give them a good Cathartic of C. C. Pills, or if they are not old enough to swallow pills, I give a dose of Calomel, Rhubarb and Jalap; I then make a saturated solution of Nitate of Silver, and apply it to the ulcerated tonsil twice a day with a swab. If the breath is much fœtid, I take a tea spoon full of the Chlorate of Potash and disolve in half a tea cup of water, and wash the mouth and throat with that three or four times per day, but never fail using the Nitrate of Silver twice a day until the ulcer disappears. If there is much fever, after the bowels are well moved, I give a powder every three hours, composed of Dover's Powder, Nitrate of Potash and Jame's Powder. I have the throat extremely well bathed in a mixture of camphor, turpentine and lard, and if it is much inflamed and swollen, I also apply an aconite poultice. I find that they generally get well under this treatment, in from two, to four days.

Yours Very Truly,
GEO. W. SLACK.

---

### CAFFEINE &c.

Our own experience fully confirms the following editorial from the Aug. No. of the *Southern Medical and Surgical Journal*, in relation to the use of Caffeine in counteracting the effect of Opium—and we have also found it very valuable as a nerve excitant in various conditions of disease, especially in the stage of exhaustion from attacks of Cholera and Cholera Infantum:

CAFFEINE IN OPIUM-COMA. *The Second Case of the Injection of Caffeine, by the Rectum, in Extreme Narcotism of Opium.* By HENRY F. CAMPBELL.

"In the May number of the *Southern Medical and Surgical Journal*, of the present year, we reported the particulars of a case of Opium-Coma, of a very grave character, in which *twenty grains of Caffeine*, injected into the rectum, produced the most surprising and satisfactory results. At the close of that former paper, we expressed the wish that some member of the Profession would repeat the treatment applied by us in that case, and either confirm or disprove our confidence in the remedy. The various medical journals of the country have commented upon the paper, and have generally approved the rationality of the measure, but, as yet, we have not been gratified by observing the report of any second trial of Caffeine under the circumstances, or any additional evidence in support of our favorable conviction in regard to the antidote. A case which occured to us on the 10th of July instant, affords us the privilege of being able to report the second case of the application of Caffeine for Opium-Coma. Although the following case was not attended by the same happy results as that reported in our May number, we think that the details of the phenomena, so far from weakening our confidence in the remedy, will go far to confirm it.

July 10th, 1860, 3½ o'clock, P. M., called in haste to the U. S. Hotel, in this city, to visit a gentleman, said to have been found in a dying condition in one of the rooms. The patient was Mr. Moses Pike, aged about 28 years, of good constitution apparently, and well developed corporeally. On entering the room, we found him in the following condition: He was entirely unconscious; face of a dark purple hue; hands and feet also purple from congestion; nails on fingers and toes of an indigo color. There was also patches of venous congestion, presenting a darkened hue all over the surface. His respiration was fearfully slow when counted, *not quite four to the minute.* The attendants were slapping and shaking him each time between the inspirations, to excite him to breathe. His respiration seemed greatly obstructed by the accumulation of mucus. Pulse very feeble, and about 100 per minute. The muscular system was completely relaxed, so that his head would fall about by its own weight, and his arms and legs obeyed only the influence of gravity.

Immediately on our arrival, a paper was found, on which the unfortunate man had recorded the fact that he had taken laudanum at 12 o'clock the night previous, with the intention of self-destruction. Two empty vials, labelled laudanum, one of two ounce capacity, the other of one ounce was found on the table. One of these vials had the neck knocked off, apparently with the view of opening it hastily—and some of the laudanum had escaped so as to leave a stain upon the label. It is probable, therefore, that the entire three ounces had not been taken. Once or twice during the morning, the servant stated, that he had approached and tried the door, with the view of entering, but had desisted when he heard the occupant snoring deeply, as he did not wish to disturb him. Somewhat after 3 o'clock P. M. the servant became alarmed and looked into the room through the transom-light from a chair, and observing his condition, called for assistance.

From the above circumstances, as well as from the written statement of the patient, it was highly probable that near 3 ounces of laudanum had been in his system nearly fifteen hours—that so large an amount had not produced death in so long a time, is truly unaccountable.

The condition of the patient, the necessity of constantly provoking respiration, and also the little probability that any laudanum yet remained in his stomach, caused us to abandon the idea of using the stomach-pump. Emetics of course were out of the question, and we at once resorted to the application of ice to the scalp, and pouring ice-water, from a distance, upon the head, while we sent for a drachm of Caffeine, and a small syringe. As soon as these arrived, we poured out in the palm of the hand what we supposed to be about twenty grains of Caffeine, desolved it in two ounces of cold water, and introduced it into the rectum by means of the syringe.

The syringe being small, three applications were made at short intervals. The whole of the alkaloid was not dissolved. By an estimate made subsequently, calculating what had been lost, the patient had taken near *twenty-five grains of Caffeine* in the three applications.

The Caffeine was administered at twenty minutes before four o'clock, at which time, as we have said, the respiration of the patient was *scarcely four to the minute*, and constant efforts were necessary, in the way of slapping and shaking to provoke him to inspire. At fifteen minutes after four, (35 minutes after the injection) his respiration was found to be effected with less effort and more regularly—and, on counting it by the watch, it numbered eight to the minute. The skin, even now, began to present less of the cerulean tint. In one hour after, the respiration had risen to twelve, and shortly rose to sixteen to the minute, when the skin was nearly of the natural hue, though the nails on both hands and feet remained still of a purplish cast.

Slight spasmodic movements in the fingers were now observed, and also some occasional subsultus in the muscles of the forearm—the under lip, which before was hanging, now became elevated and slightly compressed against the teeth. When the hand of the patient was held, and an attempt made to extend the arm at the elbow, decided muscular resistance was observed. The lid of the left eye was also observed to be raised and let down rapidly once or twice.

The pulse had now become full and somewhat resisting, and the action of the heart, as observed at the chest, tumultous. On being raised, the patient, once, made a noise slightly resembling a groan, but from the beginning to the end, he did not once manifest the least consciousness.

For a short time after the improvement in the respiration began, the mucous rale seemed somewhat to diminish, and his breathing, were it not for a certain jerking, resembled very nearly a man in deep, healthy sleep. The rale now, however, (half-past 7 o'clock) became more and more obstructive, the gurgling reaching up into the throat and threatening momentarily to strangle the patient. It was now plain that he could not survive, and, on turning him upon the right side, a bloody mucus bubbled out of the nostrils. The number of the respirations was at this time twenty to the minute, when counted by the watch. The entire surface of the body was intensely hot and remained so to the time of the patient's death, which took place about 15 minutes before nine o'clock, P. M. He seemed to die from the accumulation of the bloody mucus, in the bronchial tubes and larynx. During the whole time, from the first moment of our seeing him till the time of his death, the application of ice was made constantly to the head of the patient, and also mustard plasters were applied to the spine and to the extremities.

A superficial glance at the foregoing case might perhaps impress the reader with the conviction that the confidence which we expressed, in our former report, in Caffeine as an antidote in Opium Coma, was somewhat hasty and misplaced. A more deliberate consideration, however, will remove such an impression. When we reflect on the amount of the opium taken, the length of time during which the patient had been left to its toxic influence, and the destructive ravages which had been made during that time, we certainly, on the other hand, must feel great surprise at the amount of modification the Caffeine was seen to produce under such disadvantageous circumstances. The respiration, in a space of time, less than one hour, was raised from four to sixteen in the minute. The color of the skin, under its influence, was changed from an almost indigo hue, to that of the natural complexion, and the muscular relaxation was replaced by a fair degree of tonicity accompanied by occasional twitchings. The mode of death, too, was not such as is seen in the demise from the unmodified effects of opium, when the respiration becomes gradually slower and slower till it ceases altogether, but at the time of our patient's death, his respiration numbered twenty per minute, and he died apparently *drowned* by the accumulation of the viscid mucus in air-passages, doubtless the result of the long enduring pulmonary congestion occuring previous to the administration of the Caffeine.

In conclusion, we feel confident in saying that we feel greatly encouraged by the developments of this second case, and shall use the remedy hereafter, with even more confidence than before. We again express the hope that some of our professional brethren will add their published testimony to ours so as to establish the true amount of value that should be attached to Caffeine as an antidote in Opium-Coma.

We intend shortly reporting the results of experiments, with the two drugs, Opium and Caffeine, as made by us, on the lower animals.

---

Messrs. J. H. Reed & Co., whose card will be found on the cover of the "Examiner," have recently fitted up rooms over their store exclusively for their *Surgical and Dental Instruments*. They have been compelled to make this change owing to the increase in trade in these goods, as also the necessity of more room to exhibit properly their large stock of Instruments. They keep every thing needed by the professions, and have now a better arrangement for showing new instruments and improvements. They have arrangement with Messrs. Teimann & Co. to receive all new instruments as they are brought out, and will be pleased to show their stock whether parties wish to purchase or not.

---

LARGE CHILD.

Mrs. Wilkinson Confined July 28th, 1860. Child, female. Distance around breast immediately under the arms 15 inches; around arm 5¾ inches; around thigh 8¾ inches; around pelvis 15 inches; shortest circumference of head 14½ inches; whole length from crown of head 24 inches; weight dressed 13½ pounds. For fear some would make to much deduction for clothes, I took the precaution to have the child weighed without clothes, and it was 12½ lbs. The parents each weigh 195 lbs., and the labor terminated without operative interference.

N. HOLTON, M. D.

Buda, Ill., July 30th, 1860.

